# Regulation of Tumor Metabolism and Extracellular Acidosis by the TIMP-10–CD63 Axis in Breast Carcinoma

**DOI:** 10.3390/cells10102721

**Published:** 2021-10-12

**Authors:** Abdo J. Najy, Young-Suk Jung, Seongho Kim, Rafael Fridman, Hyeong-Reh C. Kim

**Affiliations:** 1 Department of Pathology, School of Medicine, Wayne State University, Detroit, MI 48201, USA; anajy@med.wayne.edu (A.J.N.); youngjung@pusan.ac.kr (Y.-S.J.); rfridman@med.wayne.edu (R.F.); 2 Department of Oncology, Barbara Ann Karmanos Cancer Institute, Detroit, MI 48201, USA; kimse@karmanos.org

**Keywords:** TIMP-1, CD-63, acidosis, CAIX, tumor metabolism

## Abstract

A hallmark of malignant solid tumor is extracellular acidification coupled with metabolic switch to aerobic glycolysis. Using the human MCF10A progression model of breast cancer, we show that glycolytic switch and extracellular acidosis in aggressive cancer cells correlate with increased expression of tissue inhibitor of metalloproteinase-1 (TIMP-1), known to induce intracellular signal transduction through the interaction with its cell surface receptor CD63, independent of its metalloproteinase inhibitory function. We found that, in aggressive breast carcinoma, the TIMP-1–CD63 signaling axis induced a metabolic switch by upregulating the rate of aerobic glycolysis, lowering mitochondrial respiration, preventing intracellular acidification, and inducing extracellular acidosis. Carbonic anhydrase IX (CAIX), a regulator of cellular pH through the hydration of metabolically released pericellular CO_2_, was identified as a downstream mediator of the TIMP-1–CD63 signaling axis responsible for extracellular acidosis. Consistently with our previous study, the TIMP-1–CD63 signaling promoted survival of breast cancer cells. Interestingly, breast carcinoma cell survival was drastically reduced upon shRNA-mediated knockdown of CAIX expression, demonstrating the significance of CAIX-regulated pH in the TIMP-1–CD63-mediated cancer cell survival. Taken together, the present study demonstrates the functional significance of TIMP-1–CD63–CAXI signaling axis in the regulation of tumor metabolism, extracellular acidosis, and survival of breast carcinoma. We propose that this axis may serve as a novel therapeutic target.

## 1. Introduction

Cancer cells evolve through genetic and epigenetic alterations in metabolic pathways that allow for their survival and proliferation in unfavorable microenvironments [1]. A classical metabolic adaptation of tumor cells is a shift from oxidative phosphorylation (OXPHOS) to aerobic glycolysis as a main source of ATP irrespective of oxygen availability, a phenomenon referred to as the Warburg effect [2,3]. This phenotype is known to promote apoptosis resistance [4,5,6], the generation of biosynthetic precursors for proliferation [1], and increased invasive ability [7]. Breast cancer is the most diagnosed non-cutaneous cancer and second leading cause of cancer death in American women [8]. Among conventional therapeutics for breast cancer is the use of genotoxic radiation and chemotherapy. Even with advancements in our understanding of the molecular basis of breast cancer, therapeutic resistance is a grim reality resulting from the cancer cell’s ability to survive therapeutic insults. As such, it is crucial for the field to understand the cell survival program within malignant tumors.

The tissue inhibitor of metalloproteinase-1 (TIMP-1) plays complex roles in cancer development and progressions [9,10,11,12,13]. TIMP-1 has two functional domains, consisting of the N-terminal metalloproteinase inhibitory and the C-terminal cytokine-like domains [14,15,16]. TIMP-1 was shown to serve as a key cytokine playing a role in creating a “chemoresistant niche” in Burkett’s lymphoma [17]. This corroborated the novel findings that our group originally made demonstrating TIMP-1 as a survival factor against cell death stimuli in breast epithelial cells [11,12,13]. Our study further identified CD63, a member of the tetraspanin family, as a cell surface receptor which interacts with the C-terminal domain of TIMP-1 [18]. TIMP-1 interaction with CD63 activates signaling pathways that promote apoptosis resistance and epithelial-to-mesenchymal transition (EMT) via TWIST induction [19]. These findings, together with clinical observations of elevated TIMP-1 levels in multiple cancers including breast cancer, established an “oncogenic” function of TIMP-1, seemingly paradoxical to the “tumor suppressive” activity of TIMP-1 thought to be mediated by its well-established ability to inhibit metalloproteinase activity (reviewed in [14,20]). Consistently, studies in breast cancer showed that high TIMP-1 levels correlate with reduced disease-free survival (DFS) and overall survival (OS) [20,21].

The carbonic anhydrase (CA) family members regulate cellular pH through transport and hydration of carbon dioxide, thereby playing a critical role in supporting tumor cell survival with increased glycolytic tumor metabolism [22]. In breast cancer, CAIX expression was shown to be a marker of poor prognosis [23]. Interestingly, CAIX levels in the serum of breast cancer patient correlated with TIMP-1 levels, which together predicted unfavorable DFS and OS [21,23,24]. Consistent with the functions of the CA family members, the transmembrane CAIX dimer catalyzes the hydrolysis of metabolically released pericellular CO_2_ to HCO_3_^−^ and H^+^. HCO_3_^−^ is transported back to the cells by Na^+^:HCO_3_^−^ cotransporters (NBC), preventing the acidification of the intracellular milieu [25,26]. As a result of H^+^ accumulation, CAIX contributes to the generation of an acidic extracellular environment, thereby indirectly promoting tumor cell migration, invasion, chemo and radiation resistance, as well as immune evasion [27]. As a classic response gene of hypoxia-induced factor (HIF1α), CAIX plays a critical role in hypoxic environments through the regulation of cellular acidosis supporting tumor cells survival [28]. 

Knowing that CAIX and TIMP-1 are involved in breast cancer cell survival and are clinically significant in predicting breast cancer patient survival, we assessed the possible interaction between TIMP-1 and CAIX as well as the impact this signaling network may have on breast cancer cell metabolism, especially a shift to glycolysis, and cell survival. Here, we demonstrated that TIMP-1–CD63 signaling induces HIF1α expression leading to a glycolytic switch as well as upregulation of CAIX expression/activity. We also showed that CAIX plays a critical role in TIMP-1–CD63-mediated breast cancer cell survival. Our findings shed light on a novel TIMP-1 autocrine signaling network critical for the regulation of tumor cell metabolism and the survival of breast carcinoma.

## 2. Materials and Methods

### 2.1. Cell Culture

Human MCF10A and its variant cells were generated within our cancer center and cultured in DMEM/F12 (Thermo Fisher Scientific, Green Island, NY, USA) containing 5% horse serum, 1% L-glutamine, 1% penicillin and streptomycin, 0.2% Fungizone, 0.5 μg/mL hydrocortisone, 20 ng/mL epidermal growth factor, 10 μg/mL insulin (Sigma, St. Louis, MO, USA), and 0.1 μg/mL cholera enterotoxin (Sigma). MCF10A cells are spontaneously immortalized non-malignant normal breast epithelial cell lines [29]. The pre-malignant MCF10AneoTs were established by H-ras transfection of MCF10A cells [30]. The malignant MCF10CA1h cell lines were derived through in vivo (trocar) and in vitro (organoids) passaging of the MCF10AneoT cells [31,32].

### 2.2. shRNA Mediated Knockdown 

Control shRNA sequence (shCont; Cat. #RHS4346), shRNA against TIMP-1 (shTIMP-1; Cat. #RHS4430-101134392), or CD63 (shCD63; Cat. #RHS4430-98513127) were obtained from Open Biosystems (Huntsville, AL, USA). To generate stable expressing cells, MCF10CA1h cells were grown to subconfluence then infected with shScrm, shTIMP-1, or shCD63 lentivirus at 3 MOI (multiplicity of infection) per manufacturer’s protocol. Infected cells were selected with 0.4 μg/mL of puromycin for 10 days, and the resulting pooled population was used for experimentation.

MCF10CA1h cells were grown to subconfluence then transfected with 4 µg of scrambled (Open Biosystems RHS4346) or two CAIX shRNA expression plasmids (Open Biosystems RHS4430-100993056 and RHS4430-100996131) using Lipofectamine 2000 (Life Technologies, Carlsbad, CA, USA) per manufacturer’s protocol. Stable transfected cells were then selected for use with 0.5 µg/mL puromycin for 10 days, and the resulting pooled population was used for experimentation.

### 2.3. Reagents and Antibodies

Anti-TIMP-1 (Clone 102D1) was purchased from NeoMarkers, Inc (Thermo Fisher Scientific). Anti-CD63 and anti-β-actin antibodies were purchased from CHEMICON (MilliporeSigma, Burlington, MA, USA). Anti-HIF1α and hexokinase II (HKII) were obtained from Cell Signaling Technology (Danvers, MA, USA), and Anti-CAIX was from AbCam (Cambridge, MA, USA). Anti-GAPDH was purchased from Santa-Cruz Biotechnology (Dallas, TX, USA).

### 2.4. Gene Expression Omnibus (GEO) Analysis

The GSE11259 [23] dataset was analyzed with the NCBI GEO2R. Expression values were then presented in a box plot as GEO calculated values.

### 2.5. Protein Expression Profile

All experiments were performed under normoxic conditions. MCF10A and its variant cells were grown to subconfluence, washed with PBS, then cultured with serum free media for 24 h. Conditioned media were collected, and cell debris was removed by spinning the conditioned media at 2000 RPM for 5 min at 4 °C. A volume of 45 µL of cleared conditioned media were loaded on a reducing 10% polyacrylamide gel. For cell lysate immunoblot analysis, cells were scraped in cold PBS then centrifuged at 2000 RPM for 3 min. Cells pellets were then lysed for 30 min in 1X RIPA lysis buffer (MilliporeSigma, Burlington, MA, USA) or SDS lysis buffer (0.5% SDS, 60 mM Tris-HCl—pH 7.5, 2 mM EDTA—pH 8.0, 2.5% Triton-X 100) supplemented with 100 mM PMSF, 200 mM NaVO_3_, 1 M NaF, and 8% 50X protease inhibitor cocktail (Roche, Indianapolis, IN, USA). Protein concentration was determined using the Pierce BCA protein quantitation assay (Thermo Fisher Scientific). A total of 50 µg of protein was loaded on a reducing 10% polyacrylamide gel. Gels were transferred onto nitrocellulose paper (Fisher Scientific, Waltham, MA, USA), blocked in 5% milk dissolved in tris-buffered saline 2% Tween-20, and probed with the respective antibody overnight at 4 °C at a dilution of 1:1000. Blots were then washed and probed with an HRP-conjugated secondary for 1 h at RT then developed using the Western Lightning Plus-ECL (PerkinElmer, Waltham, MA, USA).

### 2.6. Gene Expression Analysis 

MCF10A and its variant cells were grown to subconfluence, washed with PBS, then cultured with serum free media for 24 h. Cells were scraped in cold PBS then centrifuged at 2000 RPM for 3 min. RNA was extracted using the Qiagen RNeasy kit, and 1 µg of RNA was used to perform reverse transcription using the iScript cDNA Synthesis Kit (BioRad, Hercules, CA, USA). PCR was performed using the GoTaq^®^ Flexi DNA Polymerase mix (Promega, Madison, WI, USA) with the following primers: primers for TIMP-1, forward 5′-CACCAGAGAACCCACCATGGC-3′, reverse 5′- CACTCTGCAGTTTGCAGG-3′; HIF1α, forward 5′-ACCCACCGCTGAAACGCCAA-3′, reverse 5′-TCAGGGCTTGCGGAACTGCT-3′; CAIX, forward 5′-GGGTGTCATCTGGACTGTGTT-3′, reverse 5′-CTTCTGTGCTGCCTTCTCATC-3′; CAXII, forward 5′-CTGCCAGCAACAAGTCAG-3′, reverse 5′-ATATTCAGCGGTCCTCTC-3′; GAPDH, forward 5′-ATCACCATCTTCCAGGAGCGA-3′, reverse 5′-GCCAGTGAGCTTCCCGTTCA-3′. PCR conditions were denaturation at 94 °C for 90 s, annealing at 55 °C for 30 s, and extension at 72 °C for 150 s.

### 2.7. Glucose Uptake Assay

Cells were grown to subconfluence then serum starved in phenol-red free media. The conditioned media were cleared by centrifugation at 2000 RPM for 5 min. A volume of 50 μL was used for glucose measurement (read at 570 nm) using the colorimetric Glucose Assay Kit (BioVision Research Products, Mountain View, CA, USA) following manufacturer’s instructions. Glucose uptake was determined by subtracting the amount of glucose in each sample from the total amount of glucose in the media then normalized to cell number for each sample.

### 2.8. Measurement of Lactate Concentration

Cells were grown to subconfluence then serum starved in phenol-red free media. The conditioned media were cleared by centrifugation at 2000 RPM for 5 min. A volume of 50 μL was used for lactate measurement using a colorimetric kit (read at 450 nm) according to the manufacturer’s instructions (BioVision Research Products, Mountain View, CA, USA). Lactate concentration was then normalized to cell number for each sample. Each condition was in duplicate, and the experiment was performed twice.

### 2.9. Cytochrome c Oxidase (COX) Activity Measurements

COX activity was analyzed in a closed 200 μL chamber equipped with a micro Clark-type oxygen electrode (Oxygraph system, Hansatech, King’s Lynn, UK). Cultured cells were washed with phosphate buffered saline (PBS), harvested by scraping in the presence of 10 mL PBS, collected by centrifugation (500× *g*, 5 min), washed once more with PBS, and sonicated as described in [33]. Measurements were performed in measuring buffer (10 mM K-HEPES (pH 7.4), 40 mM KCl, 1% Tween 20, 2 μM oligomycin, 1 mM PMSF, 10 mM KF, and 2 mM EGTA in the presence of 20 mM ascorbate and increasing amounts of cow heart cytochrome C. Oxygen consumption was recorded on a computer and analyzed with the Oxygraph software version 1.0.0.1. Protein concentration was determined with the DC protein assay kit (Bio-Rad, Hercules, CA, USA). COX activity was defined as consumed O_2_ (μM)/min/total protein (mg).

### 2.10. Respiration Assay

Oxygen consumption of the cells was measured in a closed 200 μL chamber equipped with a micro-Clark-type oxygen electrode (Oxygraph system, Hansatech) at 25 °C and analyzed with Oxygraph software. A total of 500 μM KCN was added at the end of each measurement to inhibit cytochrome c oxidase. Non-cytochrome c oxidase-based respiration was subtracted to determine oxygen consumption rates. Respiration was defined as oxygen consumed (μM/min per mg protein).

### 2.11. Extracellular pH Analysis

Cells were grown to confluence, washed with warm PBS, then cultured with a HEPES-buffered phenol-red free DMEM (Thermo Fisher Scientific) for 16 h. pH was assessed as described by Najy et al. [34] where conditioned media were collected, and the cell debris was removed by centrifugation at 2000 RPM for 5 min. The pH of the conditioned media was measured using a Beckman Ф300 Digital pH Meter, and pH readouts were subtracted from the pH at time zero to determine changes in extracellular pH, which were further normalized to the live cell numbers (10^6^ cells). Extracellular pH was also monitored using a fluorescent pH indicator [35]. Parental MCF10A cells were plated in a 35 mm glass bottom dish (MatTek, Ashland, MA, USA) and grown for 16 h then loaded with the 10 µM N-(Fluorescein-5-Thiocarbamoyl)-1,2-Dihexadecanoyl-sn-Glycero-3-Phosphoethanolamine (DF) dye for 10 min. Cells were then stimulated with the conditioned media from the respective MCF10A cell variants and imaged using a Zeiss Axiovert 200 at 40× magnification. Experiments were read in triplicates and performed in at least three separate experiments.

### 2.12. Cell Survival and Apoptosis Assay

Cells were plated at the density of 2000 cells/well in 96-well plates for 24 h. Cells were then washed and cultured with HEPES-buffered DMEM for 48 h. Cells were fixed with 50% trichloroacetic acid, washed with water, then stained with a sulforhodamine B solution according to the Sulforhodamine B based In Vitro Toxicology Assay (Sigma) kit protocol. SRB was measured by absorbance at 565 nm wavelength using a BioRad Microplate reader. To detect apoptotic cell death, cells were plated in 35 mm glass bottom dishes and cultured with HEPES-buffered DMEM for 48 h, followed by the TUNEL assay using the fluorescein based In Situ Cell Death Detection Kit (Sigma). Cells were imaged using a Zeiss Axiovert 200 at 40× magnification, and quantitation was performed using 5 high powered fields per experimental condition. Assays were performed in triplicate and repeated at least 3 independent times.

### 2.13. Statistical Analysis

Statistical significance was determined using unpaired Student’s *t-*test, and differences were considered significant when *p* value was less than 0.05.

## 3. Results

### 3.1. TIMP1 Levels Correlate with a Metabolic Shift in the Breast Cancer Cell Progression Model

**Figure 1:**
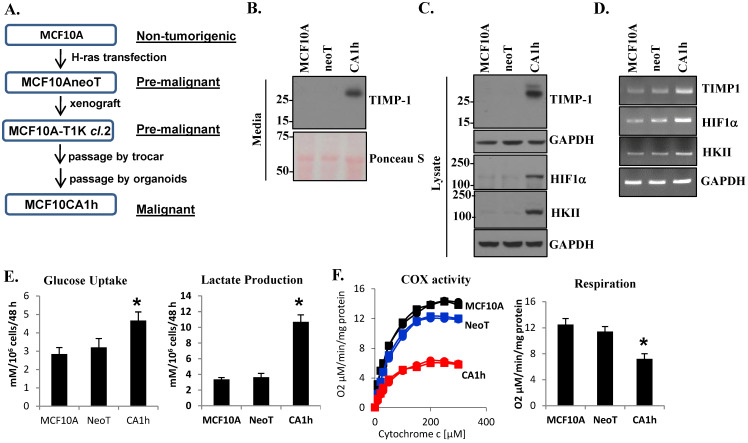
TIMP-1 expression in the human MCF10A breast cancer progression model correlates with aerobic glycolysis. (**A**) Diagrammatic representation of the development of the MCF10A progression model. (**B**) Immunoblot analysis of TIMP-1 in the conditioned media of human MCF10A and its cell line variants MCF10AneoT and MCF10CA1h. Immunoblot (**C**) and RT-PCR (**D**) analyses of TIMP-1, HIF1α, and HKII in MCF10A, MCF10AneoT, and MCF10CA1h cells. (**E**) Glucose uptake (left panel) and lactate production (right panel). (**F**) COX activity (left panel; the square and the circle designations represent duplicate sets ran for each sample) and oxygen consumption rates (right panel). Bars represent the mean ± SD. * *p* < 0.05.

A distinctive feature of cancer cells that sets them apart from “normal” non-transformed cells is a unique metabolic profile [36]. In the 1920s, Otto Warburg recognized that carcinogenesis was accompanied by a striking shift to glycolysis as the major energy-generating pathway [3]. A century later, it is still unclear if this “glycolytic switch” is an epiphenomenon or a mechanistic determinant for malignancy. To address this query, we utilized the MCF10A progression model established in our institute [30,31,32]. Through in vitro culture and in vivo passages, MCF10A, MCF10AneoT, and MCF10CA1h breast cancer cells were developed with increasing malignant potential (Figure 1A and as described in [31]). MCF10A is a non-malignant breast epithelial cell line, while the MCF10AneoT is a pre-malignant line derived by H-ras transfection into MCF10A cells [29,30]. The malignant MCF10CA1h variant demonstrated anchorage-independent growth and tumorigenesis in vivo using a subcutaneous mouse tumor model [31]. We first asked whether increased TIMP-1 expression is associated with the progression of malignancy in this model. Immunoblot analysis revealed increased TIMP-1 expression in the conditioned media and the whole cell lysate as the cell became more aggressive, particularly in MCF10CA1h cells (Figure 1B,C). TIMP-1 expression was accompanied by enhanced HIF1α and hexokinase (HK) II at protein and RNA levels (Figure 1C,D). As HKII is known to be a critical enzyme in the rate-limiting step of glycolysis [37], we analyzed glucose metabolism in our MCF10A progression model. The aggressive MCF10CA1h cells exhibited increased glucose uptake accompanied by enhanced lactate production (Figure 1E). Moreover, the MCF10CA1h cells showed decreased COX activity, critical in the mitochondrial respiratory chain (Figure 1F). Consistently, MCF10CA1h cells exhibited attenuated mitochondrial respiration as assessed by their oxygen consumption compared to the non-malignant MCF10A or the pre-malignant MCF10AneoT cell lines (Figure 1F).

### 3.2. The TIMP-1–CD63 Signaling Axis Is Critical for Breast Cancer Metabolic Switch

**Figure 2:**
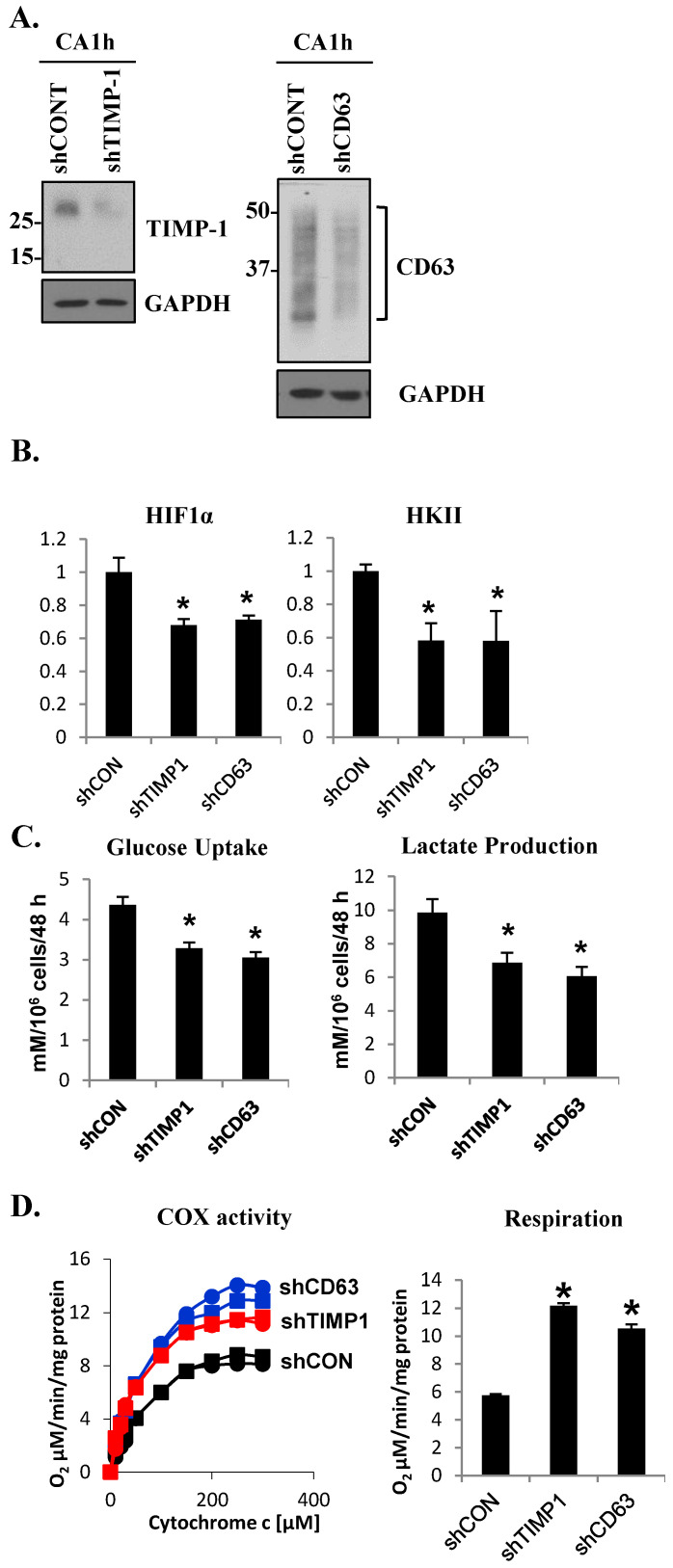
The TIMP-1–CD63 signaling axis is critical for the metabolic switch in aggressive breast cancer cell line MCF10CA1h. (**A**) Immunoblot analysis of TIMP-1 (left panel) and CD63 (right panel) in control (shCONT) and TIMP-1 knockdown (shTIMP-1) or CD63 knockdown (shCD63) MCF10CA1h cells. (**B**) Quantitative RT-PCR analysis of HIF1α and hexokinase II (HKII). (**C**) Glucose uptake (left panel) and lactate production/secretion (right panel) and (**D**) COX activity (left panel; the square and the circle designations represent duplicate sets ran for each sample) and oxygen consumption (respiration, right panel) in control and TIMP-1 or CD63 knockdown MCF10CA1h cells. Bars represent the mean ± SD. * *p* < 0.05.

TIMP-1 has been shown to possess novel signaling functions through its interaction with the tetraspanin CD63 [14,18,38,39,40,41,42]. To investigate the functional significance of this signaling complex in glucose metabolism, we downregulated TIMP-1 or CD63 in the aggressive MCF10CA1h cells using a short hairpin RNA (shRNA)-mediated gene knockdown approach (Figure 2A). Loss of TIMP-1 or CD63 led to a significant reduction in HIF1α and HKII expression as determined by quantitative PCR analysis (Figure 2B). Moreover, the disruption of the TIMP-1–CD63 signaling axis reduced both glucose uptake and lactate production in MCF10CA1h cells (Figure 2C). Conversely, the loss of TIMP-1 or CD63 resulted in increased COX activity as well as enhanced mitochondrial respiration (Figure 2D). These results demonstrated that the TIMP-1–CD63 signaling axis plays a critical role in the metabolic switch from mitochondrial respiration to aerobic glycolysis in breast cancer cells.

### 3.3. TIMP-1 and CAIX Are Co-Expressed in Breast Cancer Cells

**Figure 3:**
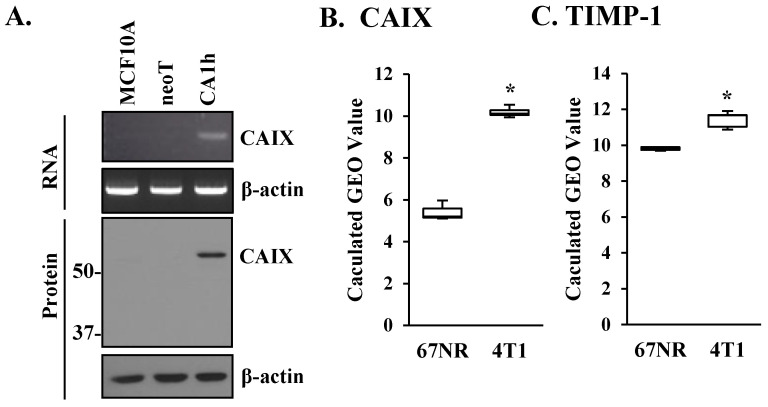
CAIX and TIMP-1 are overexpressed in aggressive human breast cancer and murine mammary cancer cells. (**A**) RT-PCR and immunoblot analyses of CAIX in MCF10A, MCF10AneoT, and MCF10CA1h cells. GAPDH and β-actin were analyzed as a control for RT-PCR and immunoblot analysis, respectively. The expression levels of TIMP-1 (**B**) and CAIX (**C**) mRNA were assessed in the microarray study GSE11295 [23] using the NCBI Gene Expression Omnibus (GEO) database. Bars represent the mean of GEO value (relative expression) ± SD. * *p* < 0.05.

As increased glycolytic tumor metabolism leads to cellular acidification, cancer cells induce the expression of pH regulators such as CA family members [22,43]. CAIX is among the most active CA family members and is also most frequently involved in human cancers, including breast cancer [25]. Although HIF1α was shown to be the exclusive regulator of CAIX expression [44,45], little is known as to what upstream signaling network activates the HIF1α–CAIX axis to cope with tumor metabolic stress. Here, we asked whether TIMP-1-induced HIF1α results in increased CAIX expression in our breast cancer progression model. RNA and protein analyses showed enhanced CAIX expression in the highly malignant MCF10CA1h cell line compared to the non-malignant MCF10A or the premalignant MCF10neoT cells (Figure 3A). These results are consistent with the previous report by Lou et al., which demonstrated a critical role of CAIX in breast cancer dissemination and observed enhanced CAIX expression in the highly invasive murine breast cancer cell line 4T1 compared to its non-invasive counterpart 67NR [23]. To further examine whether TIMP-1 expression correlates with CAIX in the 4T1 and the 67NR murine breast cancer progression models, we queried the NCBO GEO dataset GSE11259 and GEO2R [46]. CAIX was significantly upregulated in 4T1 cells compared to 67NR, confirming the findings of Lou et al. (Figure 3B). Importantly, TIMP-1 expression was also increased in the invasive 4T1 cells correlating with CAIX levels (Figure 3C), suggesting a functional relationship between the two markers as in our human breast cancer progression model. 

### 3.4. CAIX Is a Critical Determinant of Extracellular Acidification in Aggressive Breast Cancer Cells

**Figure 4:**
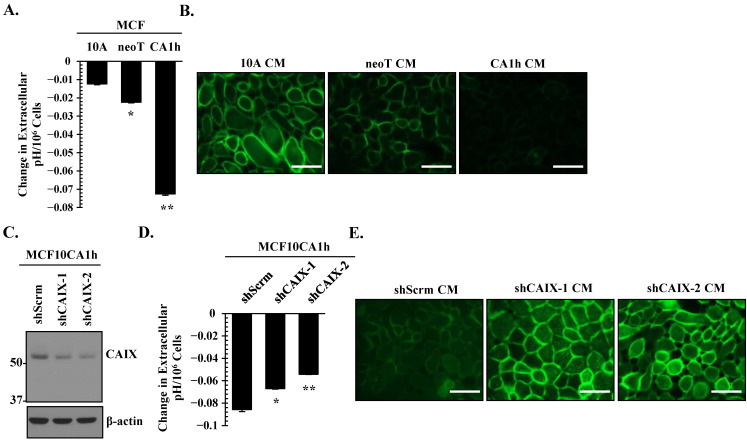
CAIX is a critical determinant of extracellular acidification in aggressive breast cancer cells. (**A**) Changes in the extracellular pH in MCF10A, MCF10AneoT, and MCF10CA1h cells were analyzed as described in the Materials and Methods. Change in extracellular pH was normalized to cell number to account for cell proliferation. Bars represent the mean ± SD. * and ** *p* < 0.05 comparing MCF10A to MCF10AneoT and MCF10CA1h cells, respectively. (**B**) Extracellular pH was assessed using the pH sensitive DF fluorescein dye. Parental MCF10A cells were loaded with the DF fluorescein dye, then stimulated with the conditioned media from MCF10A, MCF10AneoT, or MCF10CA1h cells. Scale bar represents 40 µm. (**C**) Immunoblot of CAIX in control (shScrm) and CAIX knockdown MCF10CA1h cells using two different shRNA constructs (shCAIX-1 and shCAIX-2). (**D**) Changes in the extracellular pH in control (shScrm) and shCAIX-1 or shCAIX-2 MCF10CA1h cells. Change in extracellular pH was normalized to cell number to account for cell proliferation. Bars represent the mean ± SD. * and ** *p* < 0.05 comparing shScrm to shCAIX-1 and shCAIX-2, respectively. (**E**) Extracellular acidosis was assessed using the pH sensitive DF fluorescein dye. Parental MCF10A cells were loaded with the DF fluorescein dye followed by incubation with the conditioned media from control (shScrm), shCAIX-1, or shCAIX-2 MCF10CA1h cells. Scale bar represents 40 µm.

A hallmark of solid tumors is alkaline intracellular pH (pHi) within an acidic extracellular pH (pHe) microenvironment [27,47]. This reverse pH gradient is thought to result from increased glycolytic tumor metabolism and CA activity [25]. First, we assessed whether increased CAIX expression correlates with extracellular acidosis in the MCF10A progression model. As shown in Figure 4A, we detected significant acidification within the HEPES-buffered serum-free conditioned media of MCF10CA1h cells, which correlated with CAIX expression. These findings were independent of cell proliferation, as we normalized the change in extracellular pH to cell number. We validated our numerical pH data with the extracellular DF fluorescent probe, which fluoresces more intensely under basic pH due to the unprotonated phenol and carboxylic acid functional groups [48]. This probe requires immediate fluorescent analysis (minutes after loading into cells). As a result, we were unable to monitor extracellular pH change directly on the cells of interest. To overcome this challenge, acidified conditioned media obtained from Figure 4A was placed on parental MCF10A cells pre-loaded with the DF fluorescent probe. Microscopic images revealed a decrease in fluorescent intensity in MCF10CA1h conditioned media as compared to MCF10A or MCF10AneoT (Figure 4B), indicating an acidic extracellular milieu of MCF10CA1h cells.

To assess the functional significance of CAIX in extracellular acidosis in MCF10CA1h cells, CAIX expression was downregulated using two separate shRNA constructs (Figure 4C). Attenuation of CAIX expression in MCF10CA1h cells significantly reversed extracellular acidosis, and this effect was independent of cell number (Figure 4D). Change in extracellular acidosis was validated using the DF fluorescent probe. Conditioned media from CAIX knockdown MCF10CA1h cells (shCAIX CM) demonstrated higher fluorescent intensity as compared to the control (shScrm CM), indicating more basic extracellular milieu upon CAIX downregulation (Figure 4E).

### 3.5. The TIMP-1–CD63 Axis Regulates CAIX Expression in Aggressive Breast Cancer Cells

**Figure 5:**
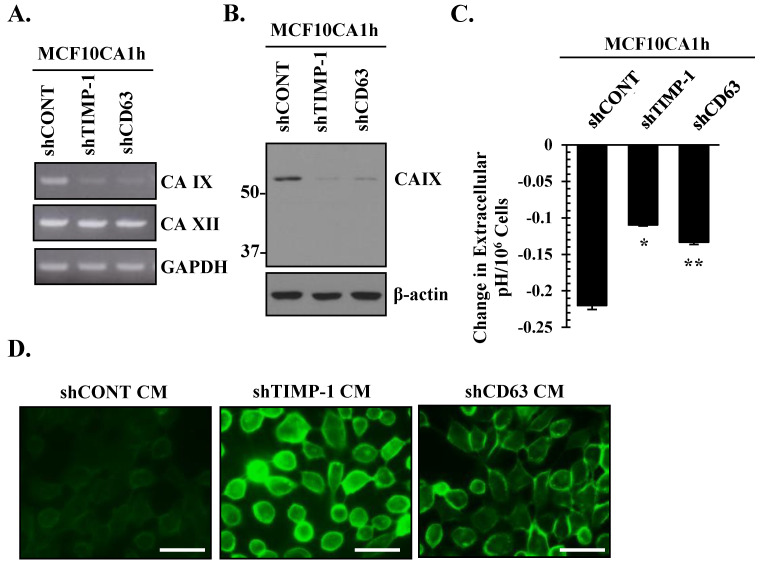
The TIMP-1–CD63 axis regulates CAIX expression and extracellular acidosis in aggressive breast cancer cells. (**A**) RT-PCR and (**B**) immunoblot analyses of CAIX and CAXII expression in control (shCONT), shTIMP-1, and shCD63 MCF10CA1h cells. GAPDH and β-actin were analyzed as controls for RT-PCR and immunoblot analysis, respectively. (**C**) Changes in the extracellular pH in control (shCONT) and shTIMP-1 or shCD63 MCF10CA1h cells. Change in extracellular pH was normalized to cell number to account for cell proliferation. Bars represent the mean ± SD. * and ** *p* < 0.05 comparing shCONT to shTIMP-1 and shCONT to shCD63, respectively. (**D**) Extracellular acidosis was monitored using the pH sensitive DF fluorescein dye. Parental MCF10A cells were loaded with the DF fluorescein dye followed by incubation with the conditioned media from control (shCONT), shTIMP-1, or shCD63 MCF10CA1h cells. Scale bar represents 40 µm.

The above analyses of breast cancer progression models showed a correlation between TIMP-1 and CAIX. Next, we examined if CAIX expression and/or activity is regulated by the TIMP-1–CD63 signaling cascade using TIMP-1 and CD63 MCF10CA1h knockdown cells described in Figure 2A. RT-PCR and immunoblot analyses revealed marked downregulation of CAIX in response to TIMP-1 or CD63 knockdown (Figure 5A,B). Importantly, the downregulation of the TIMP-1–CD63 axis did not affect CAXII (Figure 5A), indicating the specificity of the TIMP-1–CD63–CAIX signaling axis in our MCF10A breast cancer progression model. Importantly, loss of either TIMP-1 or CD63 expression abrogated the extracellular acidosis observed in MCF10CA1h cells independent of cell proliferation (Figure 5C). Consistently, parental MCF10A cells loaded with DF fluorescent probe exhibited greater fluorescent intensity upon treatment with conditioned media collected from TIMP-1 or CD63 knockdown MCF10CA1h cells as compared to conditioned media collected from control MCF10CA1h cells (shCONT) (Figure 5D). These results demonstrate the functional significance of the TIMP-1–CD63 signaling axis in the regulation of CAIX-mediated extracellular acidosis in breast cancer cells.

### 3.6. CAIX Is Critical for TIMP-1–CD63-Mediated Cell Survival in Aggressive Breast Cancer Cells

**Figure 6:**
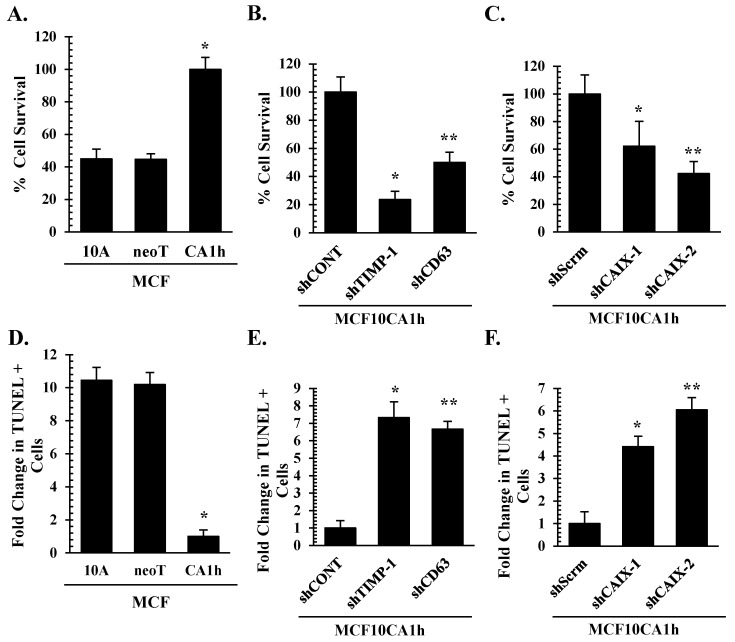
TIMP-1-mediated breast cancer cell survival is dependent on CAIX. (**A**,**D**) MCF10A, MCF10AneoT, and MCF10CA1h cells, (**B**,**E**) control (shCONT), shTIMP-1, and shCD63 MCF10CA1h cells, and (**C**,**F**) control (shScrm), shCAIX-1, and shCAIX-2 MCF10CA1h cells were assayed for their cell survival rate in serum-free HEPES buffered media for 48 h (**A**–**C**) as well as for apoptotic cell death by TUNEL assay (**D**–**F**). Read absorbance was normalized and indicated as percent cell survival or fold change in TUNEL positive cells compared to time 0. Bars represent the mean ± SD. * and ** *p* < 0.05 comparing the first bar (control) to the second and the third bars, respectively.

Increasing evidence indicates the significance of the reverse pH gradient in tumor progression [22]. While extracellular acidosis was shown to contribute to the invasive phenotype of cancer cells as well as immune evasion, alkaline intracellular pH coupled to increased glycolysis is thought to promote tumor cell survival and proliferation [23,47]. Next, we asked whether CAXI is a critical component for the pro-survival signal of TIMP-1 in cancer cells. First, we confirmed that the more aggressive MCF10CA1h cells survived significantly better than MCF10AneoT or MCF10A cells cultured in HEPES-buffered serum-free media, as determined by the colorimetric SRB cytotoxicity assay (Figure 6A), with decreased apoptotic cell death, as assessed by the TUNEL assay (Figure 6D). Loss of TIMP-1 or CD63 expression significantly reduced MCF10CA1h cell survival rate with increases in apoptotic cell death (Figure 6B,E). Importantly, CAIX knockdown significantly reduced MCF10CA1h cell survival and increased apoptotic cell death (Figure 6C,F).

## 4. Discussion

The discovery of tumor suppressors and oncogenes has stimulated cancer research at the molecular level, revealing that the phenotypic changes that are characteristic of tumor cells result from a host of mutational or deletion events that combine to alter multiple signaling pathways. It has become clear that many key oncogenic signaling pathways converge to adapt tumor cell metabolism in order to support their growth and survival. These signaling cues stimulate glycolysis by increasing the expression and the membrane translocation of glucose transporters and by enhancing key glycolytic enzymes, such as hexokinase 2 and phosphofructokinases [49,50,51]. A key regulator of the metabolic changes is hypoxia-inducible factor 1 (HIF1) [52]. Once activated, HIF1 amplifies the transcription of gene encoding glucose transporters and most glycolytic enzymes, increasing the capacity of the cell to carry out glycolysis [53]. The present study demonstrated that epigenetic changes manifested by TIMP-1 overexpression effectively induce glycolytic switch of breast cancer cells.

The tissue inhibitor of metalloproteinase (TIMPs) binds to and inhibits matrix metalloproteinase (MMPs) in a l:1 stoichiometric ratio, and the imbalance of this interaction plays a critical role in cancer progression involving MMPs-mediated tumor invasion and metastasis [54,55]. Paradoxically, however, clinical studies showed that TIMP-1 expression in particular correlates with a poor patient outcome in many cancer types, including breast cancer [10,20,56]. In addition to TIMP-1′s function as an MMP inhibitor, the cytokine-like activity of TIMP-1 is now well-established, and high levels of TIMP1 favor interactions with its cell surface receptor over metalloproteinases, leading to intracellular signal transduction [20,57]. This dynamic function can be attributed to the structure–function relationship of TIMP-1, where the N-terminus is used primarily for MMP inhibition and the C-terminus is used for protein–protein interaction [18,57,58]. To that point, our group previously identified a novel cell signaling activity of the C-terminal domain of TIMP-1 through interaction with CD63, resulting in activation of integrinβ1, FAK, PI3K, and ERKs signaling pathways for inhibition of both intrinsic and extrinsic apoptotic pathways [12,13,18,59]. Significance of these in vitro findings is supported by clinical and pre-clinical observations that elevated TIMP-1 levels were associated with a lack of response to chemotherapeutic treatment [11,60,61,62,63,64].

Extracellular acidosis is known to abrogate the effects of chemotherapeutic agents, as most are weak bases and are neutralized before they can reach their target cells [22]. In KHT sarcoma and B16F1 melanoma cells, acidosis was reported to mediate methotrexate resistance [65]. One key molecule responsible for extracellular acidosis is CAIX, whose main function in association with anion exporters is to catalyze metabolically released CO_2_, leading to increased H^+^ gradient in the extracellular milieu and alkaline intracellular pH [22,27,66]. In fact, CAIX mediates extracellular acidification of prostate, colon, and ovarian cancer cells in vitro [34,67,68] and is overexpressed in tumor tissues of colorectal, ovarian, gastric, pancreatic, and breast cancer patients [25,69]. In the current study, we made an interesting observation that increased TIMP-1 expression and its activity via CD63 correlate with malignant phenotypes of breast carcinoma using the MCF10A progression model. Ricca et al. observed a similar correlation of TIMP-1 and CD63 in their model of melanocyte malignant transformation [70]. Moreover, we found that CAIX is a critical downstream mediator of the TIMP-1–CD63 signaling axis for the induction of extracellular acidosis and cell survival. Our current findings were made in normoxic conditions, demonstrating a hypoxia-independent metabolic switch and CAIX upregulation by the TIMP-1–CD63 signaling axis. These novel findings further provided molecular insight into TIMP-1-mediated chemotherapy resistance involving glycolytic tumor metabolism and extracellular acidosis in addition to the previously characterized CD63/integrinβ1/FAK/PI3K/ERKs survival signaling pathways [12,13,14,18,59].

Outside the gastric mucosa, CAIX is undetectable in normal tissue, whereas it is highly upregulated in a multitude of cancers, especially hypoxic tumors [25,71]. This exclusive expression pattern renders CAIX an ideal target for non-invasive screening using fluorescently labeled CAIX inhibitors, which have shown great selectivity in pre-clinical trials detecting hypoxic tumors [72]. Specifically, CAIX nanobodies demonstrate effective imaging tools of pre-invasive breast cancer [73]. The TIMP-1–CD63 axis is also an attractive pathway for novel therapeutic design specifically targeting the C-terminal domain of TIMP-1 [59]. CD63 might also serve as a target using a CD63-specific monoclonal antibody such as FC-5.01, which showed great specificity to malignant tissue compared to normal tissue [74]. We propose that uncoupling the TIMP-1–CD63 signaling cascade would reverse the glucose metabolic switch and the CAIX-mediated acidosis in malignant tumor tissues in addition to inhibition of intrinsic and extrinsic apoptosis pathways. This would have additional effects on cancer cell responses to chemotherapy by preventing the neutralization of conventional therapeutics in acidic tumor microenvironment, thereby allowing chemotherapeutic agents to reach the cancer cells.

In this report, we demonstrate a novel function of the TIMP-1–CD63 signaling axis in the regulation of tumor metabolism and CAIX expression/activity, critical for breast carcinoma survival. We propose that targeting TIMP-1–CD63–CAIX may improve the therapeutic efficacy in breast cancer patients.
